# A Protocol for Developing a Staffing Prediction Model for Neonatal Care Using Time-Motion Data

**DOI:** 10.3390/healthcare14172791

**Published:** 2026-09-01

**Authors:** Kim Gibson, Maeve Downes, Wendy Duncan, Nicole Gould, Wendy Foster, Adrian Esterman, Marion Eckert, Rachael Yates

**Affiliations:** 1 Rosemary Bryant AO Research Centre, College of Health, Adelaide University, Corner of North Terrace and Frome Road, Adelaide, SA 5000, Australia; marion.eckert@adelaide.edu.au; 2 Northern Adelaide Local Health Network, Lyell McEwin Hospital, Haydown Road, Elizabeth Vale, Adelaide, SA 5112, Australia; maeve.downes@sa.gov.au; 3 Women’s and Children’s Local Health Network, Women’s and Children’s Hospital, 72 King William Road, North Adelaide, Adelaide, SA 5006, Australia; wendy.duncan@sa.gov.au (W.D.); rachael.yates@sa.gov.au (R.Y.); 4 Southern Adelaide Local Health Network, Flinders Medical Centre, Flinders Drive, Bedford Park, Adelaide, SA 5042, Australia; nicole.gould@sa.gov.au; 5 Australian Nursing and Midwifery Federation, 191 Torrens Road, Ridleyton, Adelaide, SA 5008, Australia; wendy.foster@anmfsa.org.au; 6 School of Public Health, Adelaide University, Corner of North Terrace and Frome Road, Adelaide, SA 5000, Australia; adrian.esterman@adelaide.edu.au

**Keywords:** neonatal unit, nurses, midwives, staffing, workload, infant acuity, time-motion, protocol

## Abstract

Determining appropriate nurse and midwife staffing levels in neonatal units is essential to provide safe, effective, and high-quality care to premature and critically ill infants. Ratio-based staffing methods currently utilized to guide staffing decisions consider factors such as infant acuity, specific illnesses, and treatment needs. However, these approaches may not adequately reflect contemporary neonatal care and are supported by limited empirical evidence. This study aims to generate robust evidence on the time required to provide care for infants with varying acuity levels and to develop a novel neonatal staffing model based on observed workload. This multi-site time-motion study will use one-to-one continuous observations of nurses and midwives providing care to infants across different acuity levels in South Australian neonatal units. Independent observers will record all activities performed and their duration. Descriptive statistics will be used to summarize activity characteristics, time allocation across care tasks, and infant acuity distributions. Regression modelling, including quantile and generalized linear models, will examine relationships between staffing, infant acuity, and time required for care activities. Model development will incorporate infant characteristics and nurse/midwife qualifications to estimate staffing requirements under different acuity scenarios. Sensitivity analyses and validation procedures will be undertaken to assess model reliability and generalizability. Continuous-observation time-motion studies are considered the gold standard for measuring healthcare activity duration and quantifying nursing and midwifery workload. This study will contribute to the development of an empirically informed neonatal staffing model in Australia, providing contemporary evidence to support workforce planning and to better align staffing requirements with infant care needs and acuity.

## 1. Introduction

Neonatal nurses and midwives are specialist clinicians who have a unique set of clinical skills, knowledge, and professional attributes to care for premature or critically unwell infants and their families in the neonatal unit [1]. Close infant monitoring is necessary to recognize and respond to deterioration early, to slow progression of disease, and to prevent infant death [2]. Moreover, the expertise of staff must match the needs of the infant to foster safe, high-quality care [3].

Both in Australia and internationally, gaps exist between supply and demand for nurses and midwives who have the skills and knowledge to work in neonatal units [4,5]. The gradual increase in the number of infants admitted to neonatal intensive care units (NICUs) in Australia (3.6% of all live births in 2023 compared to 3.1% in 2019) further compounds this [6]. Patient acuity levels, safety culture and missed patient care contribute to nurse/midwife moral distress, job dissatisfaction and workforce retention problems [7].

When assigning staff to care for infants, careful consideration is required to ensure that every infant has their needs met effectively and safely. Current staffing methodologies for nurses and midwives working in public-sector neonatal units in South Australia (SA) [8] are based on nurse/midwife-to-patient ratios. Ratio-based staffing methods consider factors such as patient acuity, specific illnesses, and treatment needs and assume that the number of infants assigned to a nurse/midwife adequately represents nursing workload. Patient acuity tools used across a variety of healthcare contexts have been utilized to determine staff-to-patient ratios and assume that increasing patient acuity reflects increasing nursing/midwifery workload and care time requirements, although this relationship is not consistently linear [9]. Several studies have demonstrated that nurse/midwife-to-infant ratios in NICU vary substantially with patient acuity, indicating that a fixed ratio alone does not adequately represent workload [10,11]. A neonatal acuity tool (NAT) was developed in the United States (US) and content validity and inter-rater reliability were established [3]. Barnett, Crawford and Stark [12] modified the NAT for the Australian context and demonstrated high content validity and inter-rater reliability. Further, they compared infant acuity scores with nurse/midwife-patient ratio allocations to test its performance. Infants nursed with the highest nurse/midwife-patient staffing ratio had the highest acuity score, yet agreement between acuity and ratios varied between those receiving different modes of non-invasive respiratory support. This suggests that staffing needs cannot be solely defined using a single parameter such as mechanical ventilation. Further, another consideration is the validity of acuity tools which may be compromised as it has been demonstrated that staff spend more time completing electronic medical documentation than delivering patient care [13,14]. In one study, nurses working in an American emergency department were observed to spend 30% of their shift completing electronic medical records [13].

Time-motion studies involving continuous observation are now considered the gold standard approach to collect time/duration data in healthcare. Moreover, they are considered the most rigorous strategy to quantify nursing/midwifery workload [15,16]. This methodology has contributed to understanding how nurses/midwives spend their time delivering patient care in different settings [17,18,19,20,21], providing insight into scope of practice, identifying missed care opportunities, and determining organizational barriers to care delivery.

To the best of our knowledge, only one American study, conducted in 2016, integrated direct observations with nurse expertise at a single NICU to develop a patient classification system [22]. Yet, the generalizability of this system is limited due to differences in the neonatal services provided at single institutions (e.g., complex surgical or cardiac patients), the standard use of ancillary staff such as neonatal therapists in the US [23], recent technological advancements in neonatal care [24], and the variation in floor plan and infrastructure across units. Moreover, infants with lower acuity typically cared for in Special Care Baby Units also need to be included in the staffing model.

The overall aim of this study is to develop a model to assist in informing the nursing/midwifery staff allocation requirements in South Australian neonatal units. The objectives are to (1) measure the time taken to complete nursing/midwifery activities for infants with different levels of acuity; (2) determine the association between patient acuity and the time taken to complete nursing/midwifery activities; and (3) describe the nurse/midwife qualifications that relate to the acuity of assigned infants. Reporting of this study protocol has been informed by the Transparent Reporting of a multivariable prediction model for individual prognosis or diagnosis (TRIPOD) guideline [25].

## 2. Materials and Methods

### 2.1. Source of Data

This is a time-motion study, involving one-to-one continuous observations of nurses/midwives as they perform activities in a neonatal unit. Independent observers record each single activity along with its duration. Data relating to patient acuity based on the validated NAT [12] (Supplementary Figure S1), the use of axillary clinical support, and nurse/midwife demographics, are also collected.

### 2.2. Participants

This is a multi-site study, comprising three metropolitan neonatal units in SA. Two neonatal units provide care to low-to-high risk neonates of any gestation, some surgical conditions, and those with complex congenital conditions. The third neonatal unit has the capacity to provide care for neonates ≥ 31 weeks of gestation who weigh ≥ 1500 g at birth and stable neonates ≥ 32 weeks of corrected gestation who weigh ≥ 1350 g and have been transferred for convalescence care [26].

There are two populations to be observed in this study: nurses/midwives who work in the neonatal unit, and the infants they care for. Nurses/midwives who constitute the core staff of the neonatal unit, and infants who are admitted in the neonatal unit, will be recruited to participate. Purposive sampling will be used to ensure variability in the nurse/midwife population to be studied (for example, staff with varying years of neonatal experience). Concurrently, infants with varying acuity levels will be recruited so that the model reflects a diverse sample of infants.

### 2.3. Outcome

The primary outcome measure is the total direct care time provided to each infant over one shift (the ‘infant-shift’). This will be measured through continuous observation of the primary care nurse/midwife and ancillary staff, with the duration of each activity being recorded. Where a nurse/midwife cares for more than one infant during a shift, the time spent with each infant will be recorded separately, so that each infant-shift forms a single record in the analysis. The target quantity is the total direct care time received by the infant, expressed as person-time: where more than one clinician cares for the infant simultaneously, each clinician’s time is counted, because the model estimates the staffing (person-hours) required rather than elapsed clock time. The data collection tool records each activity against a specific infant using a start/stop function so that an activity is not counted more than once, and care delivered to an infant by clinicians who are not being shadowed is acknowledged as a limitation and addressed through the unit-level aggregation. Secondary outcome measures include (1) the association between patient acuity and the time taken to complete nursing/midwifery activities, measured using the NAT [12]; and (2) the nurse/midwife qualifications and experience that relate to the acuity of assigned infants, measured through collecting demographic data of each nurse.

### 2.4. Data Collection

A list of all possible tasks comprising both direct and indirect patient care was developed by an expert committee of neonatal nursing/midwifery leaders from each study site and circulated to neonatal staff for feedback (Appendix A). After several iterations, the committee then met in person and finalized the list until consensus was achieved.

A customized, cloud-based data collection tool was developed for use with a mobile device (iPad). The tool contains a start/stop function to time the activity. Timing can be paused and resumed if the activity was interrupted. Multiple simultaneous activities, as well as the participation of additional nurses or midwives, can be captured and recorded. Activities involving more than one infant can be accurately recorded (such as multidisciplinary discussion or handovers) by utilizing the start/stop function when specific information pertaining to the observed infant is discussed.

Two to three nurses/midwives from each study site will be recruited as research observers and trained to use the tool. Inter-rater reliability testing will be conducted to ensure consistency in its application between observers on a purposive sample of neonates. Agreement between observers will be assessed in two ways: first, whether the same activity is recorded, using percent agreement and Gwet’s AC1 (preferred over Cohen’s kappa because many activities are uncommon); and second, the recorded duration of activities, using the intraclass correlation coefficient and a Bland–Altman plot. AC1 of 0.8 or above and an intraclass correlation coefficient of 0.75 or above will be considered acceptable.

Nurses/midwives who provide their consent will be observed over their allocated shifts, shadowed by the research observer. Nurse/midwife participants may alter their behaviour because they are being observed, a phenomenon known as the Hawthorne effect. To minimize its influence on staff behaviour, the observer will be a colleague known to the participant, such as the unit’s Clinical Educator. An unobtrusive electronic data collection tool will be used, and repeated observations throughout the shift will help promote natural behaviour. Demographic data of the nurse/midwife (age, years of experience, professional qualifications) will be collected to summarize the population being studied and to analyze the influence of nurse/midwife demographics on the acuity of the infant to whom they are allocated to provide care. Additional data relating to ancillary support (for example, assistance from other clinicians, organizational structures such as a dedicated emergency response team, technical support for newborn hearing screening and availability of nutrition and feed preparation) will be collected to describe site-specific nuances that need to be considered when applying the staffing model.

The observer will collect infant demographic data, including birth gestation, birth weight, current age, and the reason for admission. These data will be used to describe the infant population of the study and to articulate the variability in the population group. The participant nurse/midwife will complete the NAT [12] for the infant at the beginning and end of the observed shift to determine the association between patient acuity and the time taken to complete nursing/midwifery activities.

The observation period will be conducted over eight weeks. Each observation session will be 8, 10, and 12 h in length dependent on the time of day or rostering schedules to reflect a standard shift at the study sites. Each observer will observe one shift per day. Nurse/midwife participants will be observed over a 24 h roster (early, late and night duty), from Monday to Sunday, to reflect potential differences in staffing levels, access to specialists, and ancillary support services during after-hours periods. Across the three sites, two to three trained observers per site (six to nine in total), each observing one shift per day, will provide the capacity to record well over 80 shifts within the eight-week period. In practice, approximately 27 shift observations per site (roughly three to four per week) are required and will be scheduled across early, late and night shifts and all days of the week, leaving an ample margin for observer availability, shifts for which consent is not obtained, and the inter-rater reliability sessions. Observer fatigue will be minimized by rotating researchers at the site, using a structured electronic data collection tool with predefined activity categories, and incorporating regular breaks covered by another observer. Infant privacy and confidentiality will be maintained during observation through the use of an unobtrusive data collection tool and adherence to standard privacy practices within the neonatal unit, such as speaking quietly at the bedside and using privacy screens.

### 2.5. Predictors

Three core predictors were determined a priori based on clinical judgement rather than by automatic selection during statistical analysis, which can be unreliable and inflate optimism in small datasets. These predictors are as follows: (1) infant acuity (NAT score) at the beginning and end of the observed shift; (2) primary nurse/midwife seniority defined as a neonatal specialization qualification and five or more years of neonatal experience [11]; and (3) observed shift length (in hours).

### 2.6. Sample Size

The sample size is based on the number of observations needed to develop a stable and reliable regression model, rather than on estimating a single proportion. The primary model will relate direct care time to a small number of predictors, principally infant acuity (NAT score), with nurse/midwife seniority and observed shift hours included as adjustment variables. The study will observe 80 shifts across the three sites, with approximately 80 nurses/midwives observed during one shift. The unit of observation is the infant-shift (one infant observed over one shift), because a nurse or midwife may care for up to four infants at once. These 80 shifts are expected to yield approximately 150 to 200 infant-shift records, comfortably exceeding the number required for the model. As nurse/midwife seniority (neonatal specialization qualifications and years of experience) and observed shift hours are recorded at the nurse/midwife level, these predictors are supported by the number of staff observed (approximately 80), which also provides an adequate number of clusters for the mixed-effects analysis. Because the outcome (direct care time) is continuous, the definitive sample size follows the criteria of Riley et al. [27] for continuous-outcome prediction models, implemented in the pmsampsize command in StataNow MP 19.5; these criteria target a uniform shrinkage of at least 0.90, minimal optimism in the model R-squared, and precise estimation of the residual variance, and will be re-run once pilot estimates of the mean and standard deviation of direct care time are available. To limit overfitting given the modest sample, the number of candidate predictors will be capped a priori (approximately four to six parameters), coefficients will be estimated with penalisation or a uniform shrinkage factor, and optimism in model performance will be corrected by bootstrap internal validation.

### 2.7. Missing Data

The customized data collection tool is designed to minimize missing data through mandatory fields and real-time entry, so little missingness is anticipated. Nonetheless, the amount and pattern of missing data will be reported for each variable. Where data are missing for only a trivial proportion of records and appear consistent with a missing-completely-at-random mechanism, a complete-case analysis will be used. Where missingness is more than trivial, multiple imputation by chained equations will be applied under a missing-at-random assumption, with the imputation model including the predictors and the outcome, and estimates combined across imputations using Rubin’s rules. The primary analysis will not impute the outcome (direct care time). Any excluded records, and the reasons, will be reported in accordance with the TRIPOD guideline [25].

### 2.8. Statistical Analysis Methods

Descriptive statistics will summarize the characteristics of observed nursing/midwifery activities, time spent on various care tasks, and distributions of infant acuity. Regression modelling including quantile and generalized linear models will be employed to determine the association between nursing/midwifery staffing, patient acuity levels (as measured by the NAT [12]), and time required to complete care activities. Where an activity is delivered by more than one nurse/midwife concurrently, each clinician’s recorded time is summed, so that the activity’s contribution to total direct care time reflects the combined staff time (person-minutes) required to deliver it. Models will account for the clustered structure of the data (repeated observations within nurses and within infants) using mixed-effects (random-intercept) models or cluster-robust standard errors, with study site included as a fixed effect given the small number of sites. As activity durations are right-skewed and many task-specific times are zero, the distributional family and link will be specified (for example a gamma or log-link model, or a two-part model for activities that are frequently not performed) rather than a generic linear model. Model development will incorporate infant characteristics and nurse/midwife qualifications to predict staffing requirements across different acuity scenarios. To translate these estimates into staffing requirements, the predicted care time for each infant will be summed across all infants present on the unit during a shift to determine the total direct care time required. An allowance for indirect and unit-level activities (such as documentation, handover and restocking) and for staff breaks will be added, informed by the proportion of indirect time observed in this study. The total time required will then be divided by the direct care time one nurse or midwife can provide during a shift to estimate the number of staff required, and the relevant skill mix will be applied. Sensitivity analyses and validation approaches will be undertaken to assess model reliability and generalizability. Given the small number of sites, internal validation (for example, bootstrap resampling with optimism correction) is more feasible than external validation, and model development and reporting will follow the TRIPOD guideline [25].

#### 2.8.1. Model Development

The NAT score will be analyzed as a continuous variable, with departures from linearity examined using restricted cubic splines (or fractional polynomials) and the functional form reported. Where any data-driven selection or shrinkage is required, penalized regression (LASSO or elastic net) will be used so that coefficient estimation and variable selection are performed simultaneously. Because direct care times are right-skewed with many zero task-specific durations, models will use an appropriate distributional family and link (for example a gamma or log-link generalized linear model, or a two-part model for activities frequently not performed) rather than ordinary linear regression, and quantile regression will be used to characterize the upper tail of care time. The clustered structure (repeated infant-shifts within nurses and within infants) will be accommodated with mixed-effects (random-intercept) models or cluster-robust standard errors, with site as a fixed effect.

#### 2.8.2. Model Performance

Predictive performance will be assessed by calibration (calibration slope, calibration-in-the-large, and observed-versus-predicted plots), explained variation (optimism-adjusted R-squared), and predictive accuracy (root mean squared error and mean absolute error). Optimism in these measures will be corrected using bootstrap internal validation (approximately 500 to 1000 resamples), from which a uniform shrinkage factor will also be derived and applied to the final coefficients. Given the small number of sites, internal validation is more feasible than external validation. Model specification, performance and validation will be reported in accordance with the TRIPOD guideline [25].

## 3. Discussion

### 3.1. Limitations

A challenge in developing a generalizable nursing staffing model is the influence of organizational and contextual factors such as unit layout, workflows, and local clinical practices, which can affect workload, and the implementation of staffing systems across different healthcare settings [28,29]. By describing these factors across the study sites during model development, readers will need to consider the transportability of the final prediction model to other neonatal units. Further, aspects such as continuous clinical surveillance, anticipation of patient deterioration, and cognitive workload, which may occur concurrently with other observable activities, may not be adequately captured by the model.

### 3.2. Implications

Our planned study represents the first to develop a staffing model specific for neonatal care in Australia based on rigorous methodology for classifying nursing workload. A staffing model that is based on direct clinical observation will inform future allocations of staff to infants are appropriate to meet patient demands while avoiding overstaffing or underutilization of resources. This balanced allocation reduces costs while maintaining high-quality care. When a staffing model aligns with patient needs, it allows nurses and midwives to spend more time with each patient, providing safe and effective care. This may prevent medication errors, incidents, missed care, adverse health outcomes and increased mortality, which are observed when staffing levels do not reflect what is necessary to provide this care [30,31].

Piloting and evaluation of the staffing model will need to be undertaken prior to its implementation into practice [30]. This will require a combination of quantitative and qualitative methods to assess patient, workforce, and operational outcomes, including the usability and acceptability of the model among nursing, midwifery, and management staff. Evaluation should also include a review of staffing numbers, the use of agency staff, overtime expenditure, staff deployment to other areas, and unfilled shifts. In addition, the model’s impact on patient outcomes, including unplanned extubation, medication errors, infection rates, clinical deterioration, and length of stay, should be assessed through comparison with historical data. Future work should also include how the staffing model is perceived by parents.

## Data Availability

No new data were created or analyzed in this study. Data sharing is not applicable to this article.

## Supplementary Materials

The following supporting information can be downloaded at https://www.mdpi.com/article/10.3390/healthcare14172791/s1, Figure S1: Neonatal acuity tool [12].

## Abbreviations

The following abbreviations are used in this manuscript:

NICU	Neonatal Intensive Care Unit
NAT	Neonatal Acuity Tool

## Appendix A

**Table A1:** Nursing/midwifery task list.

Body System	Category	Activity
Respiratory	Ventilation	Set up ventilator
		Assist with intubation (including set up)
		Retape ETT
		ETT suction
		NO set up
		NO cylinder change
		IPPV bag and mask (or t-piece)
		Extubation
		Surfactant (preparation and administration)
	CPAP	Set up (including application)
		Nasal prong application/PAC
	High flow	Set up
		Nasal/oral suction
	Other	Low flow oxygen set up
		Chest drain insertion (including set up and assistance)
		Nebuliser (set up and delivery)
Cardiac		ECG lead application
		SpO2 application/repositioning
		12-lead ECG
		Defibrillation/cardioversion assistance
Vascular access		Peripheral IV insertion set up
		Peripheral IV insertion (by nurse)
		Peripheral IV insertion (assistance)
		PICC insertion set up assistance
		PICC insertion assistance
		UVC and/or UAC insertion set up
		UVC and/or UAC assistance
		Peripheral arterial line insertion set up
		Peripheral arterial line insertion assistance
		Line flushing/heparin lock
		Re-taping
		Line preparation (including sterile fluids and equipment)
Medications		Oral preparation
		Oral administration
		IV preparation
		IV administration
		IM preparation
		IM administration
		Rectal administration
		Eye drops
Transfusions		Blood transfusion
		Exchange transfusion
Clinical assessment		Set of observations (NICU) Set of observations (SCBU)
		NAS observations
		Wound/neurovascular observations
		Apnea and bradycardia management (i.e., stimulation, repositioning)
Nutrition		Milk feed preparation
		Bottle feed (complex, i.e., reflux, slow feeder)
		Bottle feed (non-complex)
		Gavage feed (<20 mL)
		Gavage feed (>20 mL)
		Nurse assist breast feeding (incl education)
		Perfusor feed set up
		Insert naso/orogastric tube
		Feeding tube pH testing
Blood sampling		Heel prick
		Arterial stab (assistance)
		Veno-puncture (by nurse)
		Arterial line
		During IV insertion
		Blood sample analysis
Procedures		Phototherapy set up
		Eye examination assistance
		Wound care
		Lumbar puncture assistance
		Urine testing
		Fecal testing
		Hearing test
		Assist with X-ray/US
		Swab collection
		Insert/assist with IDC
		Removal of IDC
Neurological		Application of aEEG
		Insertion of rectal/esophageal thermometer
		Neurological observations
		Set up for cooling mat
Cares		Nappy change
		Weigh nappy
		Pressure area care (including prongs)
		Head turn (ventilated infant)
		Repositioning
		Bathing
		Sponge bath
		Mouth/eye care
		Urinary catheter emptying
		Weighing
		Soothing infant (i.e., nurse cuddling)
		Change stoma bag
		Empty stoma bag
		Physiotherapy (incl peanut pillow)
Parent interaction		Parent education
		Any other interaction
Skin-to-skin		Facilitation
Indirect care		Documentation
		Medical round
		Teaching
		Meetings
		Pharmacy interaction
		Supplies (i.e., restocking)
		Housekeeping tasks, i.e., cleaning
		Research
		Shift handover
		Placing referrals
		Don/doff PPE
		Safety checks
Other		Handover Transporting/moving infant
		Footprints
		ID bands
		Cot change
		Linen change
		Off ward scan, i.e., MRI
		Checking and restocking emergency trolleys
		Staff breaks

ETT: endotracheal tube; NO: nitric oxide; IPPV: intermittent positive pressure ventilation; PAC: pressure area care; ECG: electrocardiogram, SpO2: peripheral oxygen saturation; IV: intravenous; PICC: peripherally inserted central catheter; UVC: umbilical venous catheter; UAC: umbilical arterial catheter; NICU: neonatal intensive care unit; SCBU: special care baby unit; US: ultrasound; aEEG: amplitude-integrated electroencephalography; IDC: indwelling catheter; PPE: personal protective equipment; ID: identification; MRI: magnetic resonance imaging.

## References

[1] Australian College of Neonatal Nurses (2025). Australian College of Neonatal Nurses Standards for Practice. https://www.acnn.org.au/resources-and-research/standards-for-practice.

[2] Gephart S.M., McGrath J.M., Effken J.A. (2011). Failure to Rescue in Neonatal Care. J. Perint. Neonatal Nurs..

[3] Roth C., Dent S.A., Luster M.H., Hering S.L., Bay R.C. (2023). Acuity Tools for the Antepartum and Neonatal Intensive Care Units. MCN Am. J. Matern. Child Nurse..

[4] Kenner C. (2022). Workforce and Supply Chain Issues Impacting Neonatal Nursing Care Globally. J. Perinat. Neonatal Nurs..

[5] Glover A. (2022). ‘Desperate’: Nurses Warn of ‘Severe’ Staff Shortage at Sydney NICU Ward. https://www.nine.com.au/australia-news/nsw/nepean-hospital-nicu-ward-nurses-claim-understaffing-impacting-care-20250707-p5zf2d.html.

[6] Chow S.S.W., Creighton P., Holberton J.R., Chambers G.M., Lui K. (2025). Report of the Australian and New Zealand Neonatal Network 2023. https://anznn.net/annualreports.

[7] Lowe P.L., Jakimowicz S., Levett-Jones T. (2022). Neonatal nurses’ professional quality of life: An integrative review. Collegian.

[8] South Australian Tribunal Nursing/Midwifery (South Australian Public Sector) Enterprise Agreement 2022. https://www.sahealth.sa.gov.au/wps/wcm/connect/969a24bf-c95d-4ba1-b4c7-5dd7e83d794b/Orders+and+EA.pdf?MOD=AJPERES&CACHEID=ROOTWORKSPACE-969a24bf-c95d-4ba1-b4c7-5dd7e83d794b-ojhoW3D.

[9] Oetelaar W.F.J.M., Rhenen W., Stellato R.K., Grolman W. (2020). Balancing workload of nurses: Linear mixed effects modelling to estimate required nursing time on surgical wards. Nurs. Open.

[10] Gagliardi L., Corchia C., Bellù R., Coscia A., Zangrandi A., Zanini R., SONAR Study Investigators (2016). What we talk about when we talk about NICUs: Infants’ acuity and nurse staffing. J. Matern. Fetal Neonatal Med..

[11] Rogowski J.A., Staiger D.O., Patrick T.E., Horbar J.D., Kenny M.J., Lake E.T. (2015). Nurse Staffing in Neonatal Intensive Care Units in the United States. Res. Nurs. Health.

[12] Barnett A., Crawford T.M., Stark M.J. (2024). Neonatal acuity tool-defined staffing ratios in a tertiary Australian neonatal intensive care unit. J. Paediatr. Child Health.

[13] Bakhoum N., Gerhart C., Schremp E., Jeffrey A.D., Anders S., France D., Ward M.J. (2021). A Time and Motion Analysis of Nursing Workload and Electronic Health Record Use in the Emergency Department. J. Emerg. Nurs..

[14] Pinevich Y., Clark K.J., Harrison A.M., Pickering B.W., Herasevich V. (2021). Interaction Time with Electronic Health Records: A Systematic Review. Appl. Clin. Inform..

[15] Lopetegui M., Yen P.Y., Lai A., Jeffries J., Embi P., Payne P. (2014). Time motion studies in healthcare: What are we talking about?. J. Biomed. Inform..

[16] Margadant C.C., de Keizer N.F., Hoogendoorn M.E., Bosman R.J., Spijkstra J.J., Brinkman S. (2021). Nurse Operation Workload (NOW), a new nursing workload model for intensive care units based on time measurements: An observational study. Int. J. Nurs. Stud..

[17] Abbey M., Chaboyer W., Mitchell M. (2012). Understanding the work of intensive care nurses: A time and motion study. Aust. Crit. Care.

[18] Yen P.Y., Kellye M., Lopetegui M., Saha A., Loversidge J., Chipps E.M., Gallagher-Ford L., Buck J. (2018). Nurses’ Time Allocation and Multitasking of Nursing Activities: A Time Motion Study. AMIA Annu. Symp. Proc..

[19] Michel O., Garcia Manjon A.J., Pasquier J., Ortoleva Bucher C. (2021). How do nurses spend their time? A time and motion analysis of nursing activities in an internal medicine unit. J. Adv. Nurs..

[20] Abt M., Lequin P., Bobo M.L., Vispo Cid Perrottet T., Pasquier J., Ortoleva Bucher C. (2022). The scope of nursing practice in a psychiatric unit: A time and motion study. J. Psychiatr. Ment. Health Nurs..

[21] Walsby A., Williams S., Gammon J., Best S. (2024). The reality of nursing time: How nurses spend their shifts. Br. J. Nurs..

[22] Daraiseh N.M., Vidonish W.P., Kiessling P., Lin L. (2016). Developing a Patient Classification System for a Neonatal ICU. J. Nurs. Adm..

[23] Pineda R., Lisle J., Ferrara L., Knudsen K., Kumar R., Fernandez-Fernandez A. (2024). Neonatal Therapy Staffing in the United States and Relationships to Neonatal Intensive Care Unit Type and Location, Level of Acuity, and Population Factors. Am. J. Perinatol..

[24] Taha S., Simpson R.B., Sharkey D. (2023). The critical role of technologies in neonatal care. Early Hum. Dev..

[25] Moons K.G.M., Altman D.G., Reitsma J.B., Ionannidis J.P.A., Macaskill P., Steyerberg E.W., Vickers A.J., Ransohoff D.F., Collins G.S. (2015). Transparent reporting of a multivariable prediction model for individual prognosis or diagnosis (TRIPOD): Explanation and elaboration. Ann. Intern. Med..

[26] Government of South Australia Standards for Maternal and Neonatal Services in South Australia 2020 Clinical Directive. https://www.sahealth.sa.gov.au/wps/wcm/connect/a31b8c0047feec57ad9bff21d1663cdf/Maternal+and+Neonatal+Services+Standards+in+SA+2020_CD_v3_1.pdf?MOD=AJPERES&CACHEID=ROOTWORKSPACE-a31b8c0047feec57ad9bff21d1663cdf-pUv7hI0.

[27] Riley R.D., Snell K.I.E., Ensor J., Burke D.L., Harrell F.E., Moons K.G.M., Collins G.S. (2019). Minimum sample size for developing a multivariable prediction model: Part I-Continuous outcomes. Stat. Med..

[28] Griffiths P., Saville C., Ball J.E., Jones J., Pattison N., Monks T. (2020). Safer Nursing Care Tool Study Group. Nursing workload, nurse staffing methodologies and tools: A systematic scoping review and discussion. Int. J. Nurs. Stud..

[29] Allen D., Strange H., Jacob N., Rafferty A.M. (2025). How can we optimise nurse staffing systems? Insights from a comparative document analysis of 10 widely used models and focused interpretative review of implementation experiences. Int. J. Nurs. Stud..

[30] Dall’Ora C., Saville C., Rubbo B., Turner L., Jones J., Griffiths P. (2022). Nurse staffing levels and patient outcomes: A systematic review of longitudinal studies. Int. J. Nurs. Stud..

[31] Rochefort C.M., Clarke S.P. (2010). Nurses’ work environments, care rationing, job outcomes, and quality of care on neonatal units. J. Adv. Nurs..

[32] National Health and Medical Research Council, Australian Research Concil, Universities Australia (2023). National Statement on Ethical Conduct in Human Research.

