# Supplementary material for: A Protocol for Developing a Staffing Prediction Model for Neonatal Care Using Time-Motion Data
Source: Healthcare (Basel). 2026 Sep 1;14(17):2791. doi: 10.3390/healthcare14172791 (PMC13565287; doi:10.3390/healthcare14172791)
Supplement: Supplementary file 1 [file healthcare-14-02791-s001.zip › healthcare-4464813-supplementary.pdf]

Supplementary Figure S1. Neonatal acuity tool [12]

Score	0	1	2	3	4
**Respiratory Status**	Self-ventilating	Cot oxygen<br><br>Intranasal	High flow <6lt	High flow >6lt<br>nCPAP<br>NIV-NAVA/NIPPV<br>Stable conventional ventilation	High frequency ventilation<br>Unstable conventional ventilation<br>iNO<br>24hr post-extubation
**Cardiac Status**	Nil monitoring	ECG monitoring (+/- non-invasive BP monitoring)<br><br>SpO_2 monitoring (+/- pre/post ductal)			Invasive BP monitoring (arterial line)<br>Arrhythmia requiring IV medications, defibrillation, or cardioversion.<br>Cardiopulmonary resuscitation or acute pulmonary cardiac arrest<br>Post-code/cardiac arrest for 24hr
**IV Monitoring**	Nil IV	1-2 peripheral IV saline block		1-2 IV peripheral lines<br>1-3 IV infusions	Umbilical lines /PCVC<br>>3 IV infusion
**Medications (doses)**	None	1-4 oral/other medications in 24hrs	5-8 oral/other medications in 24hrs<br>1-2 IV medications in 12hrs<br>102 infusion boluses in 12hrs	>8 oral/other medications in 24hrs<br>3-5 IV medications in 12hrs<br>3-5 infusion boluses in 12hrs	>5 IV medications in 12hrs<br>>5 infusion boluses in 12hrs<br>Inotrope or sedation infusions<br>Prostaglandin or insulin infusions
**Clinical Stability**	Stable	Transient apnoea, bradycardia, desaturations	Requires intervention every 2-3hrs	Requires intervention hourly	Frequent apnoea, bradycardia, desaturations requiring stimulation
**Nutrition**	Parent independently feeding	Perfuser feeds / Trophic feeds<br><br>Introducing breast feeding<br>Nurse assist with hand expressing.	Bottle or gavage <15min per feed (>50% oral and remainder gavage<br><br>Nurse assisted breast feeding for <30 min or 1-2 times in 12hrs	Bottle or gavage >15min per feed (<50% oral and remainder gavage<br><br>Nurse assisted breast feeding for <30 min for 3-4 times in 12hrs	Nil by mouth
**Parent Support & Education**	None	1-2hrs in 12hrs	2-3hrs in 12hrs	3-4hrs in 12hrs	>4hrs in 12hrs
**Blood Sampling**	None	Daily	12hrly	6-8hrly	1-4hrly (+/- TCM)
**Transfusions**	N/A			1 transfusion in 12hrs	>1 transfusion in 12hrs<br>Exchange transfusion
**Current Weight and/or Corrected Gestation**	>2000g and/or term	≤2000g and/or 35-37+6 weeks	≤1500g and/or 31-34+6 weeks	≤1000g and/or 27-30+6 weeks	≤700g and/or ≤26+6 weeks
**NAS Escalation**	N/A		Declining dose of oral medication in clinically stable neonate<br>NAS score average <8 (non-pharmacologic nursing care)	NAS score >8<br><br>Initiation of escalation of opioid replacement therapy	Rescue dose therapy.<br><br>NAS >12
**Procedures (nurse assist)**	N/A	Minor procedure totally <1hr<br>(IV insertion +/- x2 nurse assist, septic workup, ROP screening, IDC insertion)	Assist with procedures totalling >1hr<br>(LP, central/umbilical line insertion)	Assist with procedures totalling >1hr with additional clinical support (admission, intubation + surfactant)	Assist with procedures 1-2hrs with additional clinical support (exchange transfusion, chest drain insertion, MRI, CT. OT)
**Neuro**	N/A	Mild encephalopathy that is stable (not actively monitoring or treating)<br><br>Seizure precautions (including for NAS/withdrawal)	Mild encephalopathy that is acute requiring monitoring (+/- aEEG)<br><br>Declining dose of anti-seizure medications (nil seizures)	Moderate-to-severe encephalopathy that is stable (not actively cooling +/- aEEG)<br><br>Rewarming after whole body cooling or escalation of anti-seizure treatment	Moderate-to-severe encephalopathy that is acute and needing treatment (aEEG, whole body cooling)<br>Seizures or treatment of seizures that necessitates continuous monitoring (aEEG/EEG)
**Other**	N/A	Language barrier<br>Hypothermia management (hourly axilla monitoring)<br>Hourly neurovascular and/or neurological observations	Phototherapy (bank or blanket)<br>Wound care (PI care, stage 1 or 2 extravasation, post-operative sites)<br>Gastrostomy or IDC<br>Level 2 SGH surveillance	Phototherapy (banks and blanket) 2-4 lights<br>Major wound care (stage 3 or 4 extravasation, gastroschisis (+/- silo) post-op chest drain)<br>Ostomy care (any type)<br>Level 3 SGH surveillance	UWSD 1-2<br><br>Replogle tube to suction
**Skin to Skin**	Nil nurse assist		X1 nurse assist	X2 nurse assist	Unable to facilitate
**1:1**	N/A				Invasive ventilation<br>Bereavement/EOLC<br>Whole body cooling /aEEG for 24hrs
**1:2**	N/A				Overnight oximetry

Abbreviations: aEEG - amplitude integrated electroencephalography; BP - blood pressure; nCPAP - nasal continuous positive airway pressure; CGA - corrected gestational age; CT -computed tomography scan; ECG - electrocardiography; EEG - electroencephalography; EOLC - end of life care; FiO_2 - fraction of inspired oxygen; IDC - indwelling urinary catheter; iNO - inhaled nitric oxide; IV - intravenous; LP - lumbar puncture; MRI - magnetic resonance imaging; NIV-NAVA - non-invasive neurally adjusted ventilatory assist; NAS - neonatal abstinence score; NiPPV - nasal intermittent positive pressure ventilation; OT - operating theatre; PCVC - peripheral central venous catheter; PI - pressure injury; ROP - retinopathy of prematurity; SGH - subgaleal hemorrhage; SpO_2 - oxygen saturation; TCM - transcutaneous monitoring; UWSD - under water sealed drain.
